# Supplementary material for: Knowledge and Awareness of Obesity-Related Breast Cancer Risk Among Women in the Qassim Region, Saudi Arabia: A Cross-Sectional Study
Source: Healthcare (Basel). 2025 Jan 30;13(3):278. doi: 10.3390/healthcare13030278 (PMC11816457; doi:10.3390/healthcare13030278)
Supplement: Supplementary file 1 [file healthcare-13-00278-s001.zip › healthcare-3363027-supplementary.pdf]

S1

Patient Consent Form for Articles Containing Patient Details and/or Images

This form provides consent for MDPI to publish details and/or images from patients. It must be completed prior to publication.

Patient/representative details

Patient name: ___________________________________

If a representative is signing on the patient's behalf:

Name of patient representative: ___________________________________

Relationship of representative to patient: ___________________________________

By signing this form, I confirm that I have the authority to represent the patient and provide authorization on their behalf.

Article details

Article title: **Knowledge and Awareness of Obesity-Related Breast Cancer Risk Among Women in the Qassim Region, Saudi Arabia: A Cross-Sectional Study.**

Journal: **Healthcare** journal

Authors: **Amal Mohamad Husein Mackawy, Manal Alharbi, Mohamad Elsayed Hasan Badawy, Hajed Obaid Abdullah Alharbi.**

Declaration by the patient or their representative

I, the patient named above or the patient's representative, have read the abovenamed article in full (including text, figures, and supplementary material) and agree to its publication. I am fully aware of the implications of publication and accept any associated risk. In particular, I understand that, despite anonymization, it is possible that I (or the patient) may be identified based on the details or images contained in the article. While the authors and the publisher will make efforts to minimize this risk, confidentiality cannot be guaranteed.

I understand that the paper will be published online in open access format (using a Creative Commons CC BY 4.0 license, http://creativecommons.org/licenses/by/4.0), meaning that it can be downloaded, copied, and reused without limitation. This include any figures, tables, and

supplementary data. The primary audience for the published paper will be healthcare professionals, research academics, and students from across the globe.

The final published version may differ from the one submitted to the journal due to minor revisions, changes to style, and reformatting. Publication in the journal mentioned above is not guaranteed and will take place at the discretion of the publisher, and with permission of the Editor-in-Chief (or a qualified Editorial Board member) after a peer review process.

Signing this form does not remove any of my/the patient's statutory rights to privacy. I understand that I may revoke consent at any point prior to publication, but after publication, my consent can no longer be withdrawn.

I understand that I/the patient will receive no financial benefit or compensation from the publication of the article.

Patient and/or representative signature(s):

____

Place, date:

____

S2

STUDY QUESTIONNAIRE

You are invited to participate in this study titled:

Knowledge and Awareness of Obesity-Related Breast Cancer Risk Among Women in the Qassim Region, Saudi Arabia: A Cross-Sectional Study.

Study Procedures:

If you decide to participate in this study you will undergo the following procedures:

The data required for the research will be collected through the published questionnaire. After approval, you will choose the appropriate answer for each question in the questionnaire. Answering the question will move you to the next question. After completion, the data will be collected and analyzed reliably and systematically using reliable scientific sources and relevant previous studies.

A- The socio-demographic and lifestyle characteristics of the participants.

1-Age in years (AGE)

o 15-25.

o 26-39.

o Above 40.

2-Marital status

o Married

o Single

3-Education level (EDU)

o Middle school

o High school

o Diploma

o Bachelor

4-Family history of breast cancer

- **Yes**
- **No**

5- Body weight in kilograms (WEIGHT)

o Less than 40

o 40-60

o 61-80

o 81-100

o Above 100

6- Body mass index (BMI) kg/m²

- ***Underweight (***< 18.5)
- ***Normal (18.5-24.9)***
- ***Overweight (*****25.0–29.9)**
- ***Obese (> 30*****)**

7-How often do you exercise a week (exercise)

o Never

o Once to twice a week

o Three or more a week

8-Do you follow a healthy diet (DIET)

o Yes

o No

9-In which menstruation stage you are (menstrual)

o Menstruation

o Menopausal

o Postmenopausal

10- Is obesity hereditary in your family (inheritance)

- **Yes**
- **No**

11-*Source of knowledge regarding breast cancer.*

- **Media (TV)**
- **Reading**
- **Family/ Friends**
- **Educational lectures**
- **Internet**

B- The participant's knowledge regarding obesity and BC.

1- Knowledge about the calculation of the estimated BMI for obesity (BMI).

- **Yes**
- **No**

2- Knowledge about the risk of sex hormonal Drug consumption.

o Yes

o No

3-knowledge about weight gain drugs and the risk of breast cancer.

o Yes

o No

4-knowledge about obesity after menopause and risk for breast cancer.

o Yes

o No

C- Practice and awareness of the breast cancer annual examination and their information source.

1-Are you performing the self-examination of the breast at home

o Yes

o No

2-Awareness about how to perform a self-examination of the breast at home.

o Yes

o No

3-Performing a monthly self-examination

○ Yes

○ No

4 --Have you ever had a mammographic examination(mammogram)

o Yes

o No

5-Have you ever been made aware of the need for an annual examination? (Inform A)

o Yes

o No never

6-If you answer yes then by who (inform B)

o Doctor

o Someone else

7-Do you think there is enough awareness about breast cancer in Saudi Arabia (awareness)

o Yes

o No

Thank you for participating in the current study, you are completely free to choose whether to join the study or not. Your management plan will proceed according to the standard guidelines and will not be adversely affected by your decision.

Freedom of withdrawal:

You have the right to withdraw from the study at any time without explaining the causes.

Confidentiality:

Neither your name nor your file number will be written on any study document. Each patient will be given a code number on the study documents, so your data will be concealed from all study participants except the treating physician and the principal investigator.

S3

STROBE Checklist for Cross-Sectional Studies

Title and Abstract

Title: **Knowledge and Awareness of Obesity-Related Breast Cancer Risk Among Women in the Qassim Region, Saudi Arabia: A Cross-Sectional Study.**

1. -Indicate the study's design. Clearly stated as a cross-sectional study in the abstract and methods section.
2. **Abstract**: Provide an informative and balanced summary of what was done and what was found.
   - Includes objectives, methods (cross-sectional design, sample size, data collection method), key findings (poor knowledge of BC risk factors), and conclusion.

Background: Breast cancer (BC) is a major health concern globally and the second leading cause to women's cancer-related mortality in Saudi Arabia. Although awareness of BC risk factors has been previously examined, obesity-related BC awareness in the Qassim region is inconclusive. We aimed to evaluate knowledge and awareness of obesity-related BC risk among Saudi women in the Qassim region. **Methods**: This is a cross-sectional study with a stratified random sampling technique of 400 Saudi women randomly selected from the Qassim region through an online platform and community health centers. Online closed-ended pretested valid structured questionnaire using a Google Forms link. Categorical variables were listed as frequency and percentage. The chi-square test was used to study the relationship between the dependent and independent variables. **Results:** There is moderate to poor knowledge regarding breast cancer risk factors. It showed poor knowledge about obesity after menopause as a risk factor for BC (49%). Over half of the participants (51.0%) did not consider obesity a BC risk. Self-examination and mammogram showed moderated (59.6%) and poor awareness levels (4.75%). **Conclusion**: The findings highlight a noticeable gap in knowledge and awareness about obesity-related BC risk, as well as a limited awareness of breast self-examination and mammogram screening. These results underscore the urgent need for targeted awareness campaigns and educational programs in the Qassim region to address this critical health issue. Promoting breast self-examination practices, weight management, and regular mammogram screenings could significantly enhance early detection, improve prognosis, and reduce BC-related mortality among Saudi women in the Qassim region.

Introduction

3. **Background/Rationale**: Explain the scientific background and rationale for the study.
   - The introduction provides a thorough explanation of BC as a health concern globally and in Saudi Arabia, the rising obesity rates, and their connection to BC risk. Moreover, the awareness of obesity as an associated risk factor for BC is too poor in the Qassim region and needs to be improved.
4. **Objectives**: State-specific objectives, including any prespecified hypotheses.
   - The aim is clearly defined: to evaluate knowledge and awareness of obesity as a BC risk factor and assess screening practices.
   - This study aimed to measure the knowledge and awareness level of Saudi women in the Qassim region regarding obesity as an associated risk factor for BC and examine their practice and awareness level of BC early screening and diagnostic methods, which helps early BC

detection and better prognosis. Moreover, to increase the level of awareness regarding the impact of obesity and its relationship with the incidence of BC to gauge the society's awareness and knowledge levels about this connection hoping to shed light on the understanding of this crucial health relationship in the Qassim region, Saudi Arabia.

Methods

5. **Study Design**:
   - Cross-sectional study design from Jan 2024 to March 2024 is stated.
6. **Setting**: Describe the setting, locations, and relevant dates.
   - Conducted in the Qassim region, Saudi Arabia, with data collected through an online platform and health centers face-to-face interviews.
7. **Participants**:
   - Eligibility criteria, and the source and method of participant selection.
     - Inclusion and exclusion criteria (Saudi women aged ≥18 years, from Qassim region).
     - Stratified random sampling described.
   - Report the number of participants at each stage.

Detailed: 400 participants with inclusion of 250 from health centers and 150 from online recruitment. In the beginning, 200 participants were recruited through an online platform, responses of 150 of them were accepted but 50 respondents' responses were discarded as they were incomplete and missed some answers. 250 females were randomly collected from the community health centers in the Qassim region after gaining their informed consent. The authors provided assistance and clarification while filling out the questionnaire during data collection by face-to-face interviews.

- **Variables**: Clearly define all outcomes, exposures, predictors, potential confounders, and effect modifiers.
  - Variables include knowledge of obesity and BC risk factors, BMI, family history, source of information, and awareness of screening methods, such as breast self-examination and mammography.

8. **Data Sources/Measurement**: For each variable, give details of data sources and measurement methods.
   - Questionnaire design explained (validated, based on prior tools). BMI was calculated using weight and height data (Body weight in Kgs/height in meters ^{2})
9. **Bias**: Describe efforts to address potential sources of bias.

- Efforts include the exclusion of incomplete responses and pilot testing of the questionnaire.
- The questionnaire was validated and was previously published using the National Cancer Institute's online BCRA or the Gail Risk Assessment Tool [22]. A pilot group of 50 participants was held on 2nd Jan 2024, to validate the results of the questionnaire, and the decision was taken by the authors to

verify the suitability of the questionnaire length and type of questions appropriate. The participants answered some end-closed Multiple-Choice Questions, in 10-15 minutes.

11. **Study Size**: Explain how the study size was arrived at.

- Sample size calculation using standard formula ($n = z^2p(1-p)/e^2$). 400 participants were sufficient to give statistically significant and informative results

12. **Quantitative Variables**: Explain how quantitative variables were handled in analyses.

Data stratified by socio-demographic and lifestyle factors. Knowledge levels are categorized as poor, moderate, or good. Out of a maximum score of 100, the results were divided into three main categories, Poor (25–49%), Moderate (50–74%), and Good (75% and above).

13. **Statistical Methods**:
    - o Describe all statistical methods, including those used to control for confounding.

- Collected data were entered in Microsoft Excel 2016 and then analyzed by EPI INFO 7.
- Distribution and numerical data are illustrated as mean± SD. Categorical data are shown as frequency (percentage).

▪ Chi-square test and logistic regression described for associations.
o Describe any methods used to examine subgroups and interactions.
▪ Multivariate regression is used to assess relationships with knowledge.
o Explain how missing data were addressed.
▪ Missing responses excluded from the analysis.

- For the logistic regression model regarding BSE practice and awareness, knowledge levels related to breast cancer were added to the independent variables.
- The P-value of ≤0.05 was considered for the statistical significance.
- The results were interpreted in Microsoft Word 2016 in the form of tables and graphs.

Results

14. **Participants**: Report the number of participants at each stage.

Detailed in methods and results sections. Table 1 provides participant characteristics, and gives a full description of the socio-demographic and lifestyle characteristics of the study sample. Table 2. Provides the participant's knowledge number and percentage with the P-value of the chi-square (X^2) test regarding obesity and breast cancer.Table 3 for the Participants practice and awareness of the breast cancer annual examination and their information source with the P-value of the chi-square (X^2) test.

15. **Descriptive Data**:
    - Give characteristics of study participants (e.g., demographics).
        - Socio-demographic details provided in Table 1 (age, BMI, education, etc.).
    - Indicate the number of participants with missing data for each variable of interest.
        - Missing responses excluded as noted.
16. **Outcome Data**: Report numbers of outcome events or summary measures.

- Outcomes include knowledge levels and screening practices, with percentages for each.

17. **Main Results**:
    - Report unadjusted estimates and, if applicable, confounder-adjusted estimates.
        - Adjusted ORs presented for significant factors (e.g., age, education level).
    - If relevant, consider the use of plots to visually display the results.

Figures included (e.g., knowledge distribution).; Figure 1. The percentage of the participant's knowledge regarding obesity as an associated risk of breast cancer, n-400. BMI; Body mass index.
Figure 2. The percentage of the participant's awareness about the method of breast self-examination (n=400).
Figure 3. The percentage of the monthly performance of breast self-examination among the participants; n=400.
Figure 4. The percentage of mammograms performed by the participants, n=400.

18. **Other Analyses**: Report other analyses done (e.g., subgroup analyses, interactions).

- Logistic regression for BSE awareness and mammogram practice.

Discussion

19. **Key Results**: Summarize key results with reference to study objectives.

- Discusses poor knowledge and screening practice, linking findings to the study aim.

20. **Limitations**: Discuss limitations of the study.

- Identified limitations include small sample size, cross-sectional design, and potential response bias.

21. **Interpretation**: Give a cautious overall interpretation of results considering objectives, limitations, and prior research.

- Results interpreted in the context of previous studies and recommendations for awareness campaigns.

22. **Generalizability**: Discuss the generalizability of the study results.

- Findings applicable to Saudi women in Qassim, with cautious extrapolation to broader populations.

Other Information

23. **Funding**: Give details of funding sources and the role of funders.

- No specific funding reported.

24. **Ethical Approval**: State ethics committee approval and informed consent procedures.

- Ethical approval obtained; informed consent detailed.

25. **Supplementary Materials**: Include any additional information (e.g., full questionnaire).

- Questionnaire described but not appended; data availability addressed.
